# Real-Time Implementation and Comparative Analysis of FOC and FCS-MPCC-Based PMSM Drives for Electric Vehicles

**DOI:** 10.3390/s26123922

**Published:** 2026-06-20

**Authors:** Aydın Boyar, Ersan Kabalcı

**Affiliations:** Department of Electrical and Electronics Engineering, Faculty of Engineering and Architecture, Nevsehir Haci Bektas Veli University, Nevsehir 50300, Türkiye; aydinboyar@nevsehir.edu.tr

**Keywords:** electric vehicles, model predictive control, ANPC inverter, MPCC, PMSM, FOC

## Abstract

There is a growing trend towards vehicles powered by alternative energy sources due to the environmental pollution caused by fossil fuel vehicles. Electric vehicles (EVs) are thought to make a significant contribution to reducing environmental pollution. This study presents a performance comparison of field-oriented control (FOC) and finite control set-based model predictive current control (FCS-MPCC) methods for controlling PMSM motors, which are commonly preferred for EV applications. A multilevel ANPC inverter topology, which has a higher-quality power flow than classical two-level inverters, was preferred to power the PMSM. While the classical FOC method has a fixed switching frequency by including cascaded PI controllers and a pulse width modulation (PWM) modulator, the FCS-MPCC method determines a variable frequency-switching signal that minimizes the cost function by predicting the future current behavior of the PMSM using the mathematical model of the system. The performance comparison of FOC and FCS-MPCC methods was carried out by conducting real-time experimental studies. Both control algorithms were analyzed under variable speed and load conditions using the same motor and drive structure. Performance analysis of FOC and FCS-MPCC control algorithms was carried out in terms of speed tracking, torque, current, and harmonics. According to the results obtained, the total harmonic distortion (THD) value of the stator current was 7.03% in the FOC method, while it was 22.19% in the FCS-MPCC method. Furthermore, a comparative analysis was conducted on the dynamic performance of the two methods in different scenarios using the mean absolute error (MAE), root mean square error (RMSE), integral absolute error (IAE), integrated time absolute error (ITAE), and integral squared error (ISE) criteria. The FCS-MPCC method was observed to be superior in different speed scenarios according to these criteria. In terms of processor load, it was calculated as 17.09% in the FOC method and 63.75% in the FCS-MPCC method. This study is important for determining the control strategy of PMSMs used in EV drives.

## 1. Introduction

Environmental pollution has recently increased due to greenhouse gases such as carbon dioxide, hydrocarbons, nitrous oxide, sulfur dioxide, and methane. Factors such as the pollution caused by fossil fuels used in vehicles, increasing demand, limited reserves, and high cost have led to a shift towards alternative energy sources. Electric vehicles (EVs) have emerged as an alternative to fossil fuel vehicles in terms of reducing environmental pollution and consuming resources efficiently. It is estimated that battery-powered EVs will replace fossil fuel vehicles in transportation by 2030 [1,2,3]. EVs are environmentally friendly, efficient, and offer a higher performance compared to fossil fuel vehicles. Globally, EV technology is rapidly developing to achieve environmental pollution reduction and energy conservation goals [4].

Electric machines are configured in two distinct structures, either brushed or brushless. The brushless machines, which can be formed in asynchronous or synchronous operation, are commonly favored for EV applications. The permanent magnet brushless DC machines and permanent magnet synchronous machines (PMSMs), which belong to the permanent magnet class among synchronous machines, are very frequently preferred in EV applications. The PMSMs are used in many fields, including electric and hybrid vehicles, robotics, and aviation applications, and have an important place in electric drive systems due to their high torque density, high efficiency, and fast dynamic response [4,5,6].

In EVs, PMSMs are typically powered by voltage source inverters (VSIs) that convert DC voltage from batteries to AC voltage. The inverters are generally classified into two categories as two-level and multi-level topologies. The conventional two-level inverters are widely used in EV applications due to their simple structure and ease of implementation. However, two-level topology causes higher current ripples, torque jitter, and a higher harmonic ratio. To eliminate the disadvantages of two-level inverters, multilevel inverter (MLI) topologies have been proposed. The MLI topologies are currently attracting attention due to advantages such as high-quality output waveforms, lower harmonic ratios, and lower dv/dt ratios. Since MLIs have a staircase output voltage that is very similar to a sine wave, the harmonic distortion is significantly reduced, and the electromagnetic interference that significantly impacts system efficiency is reduced. The use of MLI topologies in EV applications can significantly contribute to the vehicle’s performance [2,7,8].

Conventional control techniques such as field-oriented control (FOC) and direct torque control (DTC) are commonly used in industrial applications for PMSM control. Although the DTC method has a simple structure and high dynamic performance, its steady-state performance is poor due to its high torque ripple and variable switching frequency. Although the FOC provides effective speed regulation and steady-state performance over a wide speed range compared to DTC, it lacks a hysteresis current controller and relies on a cascaded PI control structure. This feature can lead to disadvantages such as the need for precise adjustment of PI parameters. In the FOC method, the internal current loop plays a crucial role in generating the reference voltage that is used to obtain the switching signals [9,10,11]. Model predictive control (MPC) is currently attracting attention due to its ability to easily handle simple and multivariate problems. The MPC is a nonlinear control algorithm which is suitable for controlling PMSMs. When the PMSM is fed by a VSI, the MPC method can optimize the voltage output in real time according to the system model. Based on the method of obtaining voltage output, the MPC is divided into two categories—continuous control set MPC (CCS-MPC) and finite control set MPC (FCS-MPC). In the CCS-MPC method, the optimal voltage vector is obtained by inheriting the derivative of the cost function, and pulse width modulation (PWM) is used to generate the switching signals. In the FCS-MPC method, the voltage vector that minimizes the cost function is determined after performing the necessary calculations for all voltage vectors by taking advantage of the discrete structure of the system. In the FCS-MPC method, any PWM method may be used, and the frequency of the switching signals is variable [12,13]. The FCS-MPC method can generally be split into two classes as finite control set model predictive torque control (FCS-MPTC) and finite control set model predictive current control (FCS-MPCC), both of which are useful for PMSM control. In the FCS-MPTC method, the need for coordination in determining torque and flux weighting factors increases the complexity of parameter tuning. This affects the torque and flux regulation performance. The FCS-MPCC method, on the other hand, focuses only on current reference monitoring, making control implementation simpler and eliminating the need for weighting coordination. The disadvantage of the FCS-MPCC method is that it involves excessive computational load for each voltage vector in the control set and is not sufficiently successful in suppressing current harmonics [14,15].

Many studies on FOC and FSC-MPC-based PMSM drives exist in the literature. Gu et al. [16] proposed an MPCC strategy based on an extended control set (ECS-MPCC) to improve the control performance of the system compared to the conventional method by expanding the control set to select multiple vectors in a single control period for PMSM driven by a neutral point clamping (NPC) inverter, while Mishra et al. [9] implemented the control of PMSM powered by the two-level inverter using the field-programmable gate array (FPGA) with finite set model predictive control (FS-MPC). The performance of the control method was compared with traditional FOC. However, in this study, the PMSM was not driven by MLI topologies. Similarly, Hakamive et al. [17] introduced a modified predictive torque control (PTC) to reduce DC link voltage imbalance, torque and flux fluctuations of PMSM drives with an NPC inverter. The space vector PWM (SVM) method was used to obtain switching signals. In the study conducted by Zhu et al. [18], a modified multivector MPCC (MMPCC) method with a hybrid control set for PMSM drives was developed. The aim was to have a stable current and low harmonics. In [19], Gui et al. developed a disturbance feedback rejection control (DFRC)-based FCS-MPCC method for interior PMSM (IPMSM) used in high-speed trains. The effects of electrical parameter mismatch problems caused by magnetic disturbance faults on the FCS-MPCC method were analyzed. The aim was to achieve high-current-monitoring performance with the proposed control method in case of disturbances in electrical parameters. In [20], Ghanayem et al. developed a control structure to ensure the uninterrupted operation of the three-phase PMSM based on FOC where speed and flux control are performed independently in the case of open circuit fault (OCF) while, Wang et al. [21] introduced an online virtual voltage vector synthesis strategy to avoid current error in nine-phase open-ended wound permanent magnet synchronous motors (OW-PMSM) controlled by FCS-MPCC. It was experimentally validated that the steady-state performance was increased by 56% and the computational load was reduced by 13% compared to the classical FCS-MPCC strategy.

The proposed study is based on the simulation study carried out in [2]. This work implemented the control of a PMSM utilized in EVs by supplying it with an active neutral point clamped (ANPC) inverter and employing FOC and FCS-MPCC approaches separately. It is an experimental enhancement of a simulation study previously conducted by the authors. The previous study only performed a simulation analysis of the FOC and FCS-MPCC methods in controlling ANPC-based PMSM. The significance of this study lies in the fact that both control methods were implemented in real time using a TMS320F28379D DSP. The ANPC inverter topology, which offers lower harmonic distortion, higher output voltage quality, and lower dv/dt values than traditional two-level inverters, was selected to convert DC-AC voltage. The control of the PMSM was carried out using the FOC method with a constant switching frequency and the FCS-MPCC method with a variable switching frequency, and the dynamic performance criteria, harmonics, and processor load under the same conditions were analyzed in detail. The contribution of this study can be summarized as follows;

- PMSM drive studies are commonly performed using two-level inverters in the literature. However, the voltage required for the PMSM in the comparison of FOC and FCS-MPCC is supplied by the three-level ANPC inverter topology in this study.
- The control of the PMSM used in EVs was performed in real time using both the FOC (fixed switching frequency) and FCS-MPCC (variable switching frequency) methods, all at the same sampling frequency.
- The performance of the two separate control methods in three different scenarios created under the same conditions was analyzed in detail.
- The comparison of control methods was evaluated not only in terms of speed tracking but also in terms of MAE, RMSE, IAE, ITAE, ISE performance criteria, THD, and processor load.
- The developed FOC and FCS-MPCC algorithms were programmed using a Texas Instruments TMS320F28379D DSP development board to control the PMSM in real time. A comparison of the FOC and FCS-MPCC methods in terms of processor load was made.
- Experimental studies have shown that the FCS-MPCC method offers dynamic advantages when used to control PMSM, while the FOC method achieves better harmonic performance. The strengths and weaknesses of both methods have been evaluated using quantitative results.

The paper is organized as follows: Section 2 describes the ANPC inverter, FOC, and FCS-MPCC methods used to construct the PMSM drive. Section 3 presents a control study and performance comparison of the PMSM powered by the ANPC inverter using the FOC and FCS-MPCC methods separately. Section 4 presents the concluding remarks of the proposed study.

## 2. Materials and Methods

The PMSM drive system proposed in this study comprises three main components: the ANPC inverter, the FOC or FCS-MPCC method, and the PMSM. The PMSM is controlled using two separate control methods: FOC and FCS-MPCC. This section provides detailed information about the configuration and components of the proposed PMSM drive system.

### 2.1. Three-Level ANPC Inverter Topology

The ANPC topology offers an effective solution to the problem of uneven distribution of semiconductor losses encountered in NPC inverter configurations. Thanks to the two redundant neutral current paths in the ANPC topology, flexible configuration can be implemented to balance losses. This method optimizes loss distribution, provides voltage balancing, and increases system efficiency and power capacity. Furthermore, it allows the use of low-voltage withstand semiconductor components in high-voltage applications, as is the case with three-level NPC inverter topologies, which is a significant advantage. Due to these features, the ANPC multilevel inverter topology stands out as highly suitable for high-power and high-efficiency energy conversion systems [22,23].

**Figure 1:**
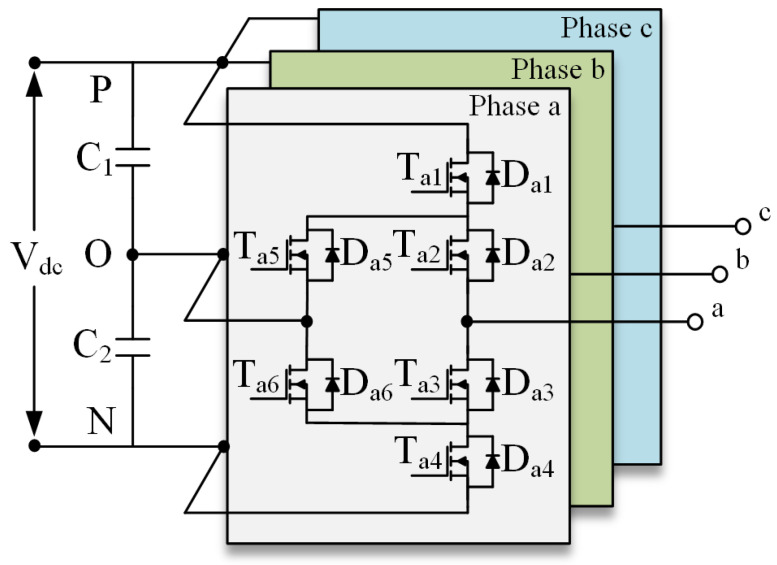
Three-phase three-level ANPC inverter topology structure.

Figure 1 shows the three-phase three-level ANPC inverter topology. In this topology, C_1_ and C_2_ are the upper and lower input capacitors, respectively, and point O represents the midpoint of the input voltage. Each phase branch has four switching elements (*T_x_*_1_, *T_x2_*, *T_x_*_3_, and *T_x_*_4_) that form the bridge structure of the inverter and perform the switching operations. Instead of the clamping diodes found in the NPC inverter structure, active switches *T_x_*_5_ and *T_x_*_6_ are used in the ANPC inverter topology. *D_x1_*–*D_x6_* represent reverse-biased diodes connected in parallel to each switching element. The ANPC inverter provides more effective neutral point voltage balancing and equalization of switching losses among phases compared to the NPC inverter topology [24].

The switching states of the three-level ANPC inverter topology are given in Table 1. Three different voltage levels are obtained at the inverter output: 0.5 V_dc_, 0, and −0.5 V_dc_, resulting in three different states: *P*, *O*, and *N*. The current paths for states *P* and *N* are obtained similarly to those of an NPC inverter. Two additional current paths connecting the output point to the neutral point are obtained by appropriately switching the switching elements *T_x_*_2_, *T_x_*_3_, *T_x_*_5_, and *T_x_*_6_. Thus, the current flow is secured more flexibly in this inverter structure. The P state occurs when switching elements *T_x_*_1_, *T_x_*_2_, and *T_x_*_6_ are in conduction and *T_x_*_3_, *T_x_*_4_, and *T_x_*_5_ are in cutoff, resulting in an output voltage of 0.5 V_dc_. The states of switching devices *T_x_*_2_, *T_x_*_5_, or *T_x_*_3_, *T_x_*_6_ play a role in determining these two current paths for the *O* state, which includes four states: *OU1*, *OU2*, *OL1*, and *OL2*. In the *OU1* or *OU2* state, switching devices *T_x_*_2_ and *T_x_*_5_ conduct, while switches *T_x_*_1_, *T_x_*_3_, and *T_x_*_6_ are in the off state. The switching state of *T_x_*_4_ determines whether the output state is *OU1* or *OU2*. When switching devices *T_x_*_3_ and *T_x_*_6_ are conducting, and switches *T_x_*_2_, *T_x_*_4_, and *T_x_*_5_ are off, the *OL1* or *OL2* zero state occurs. The state of switching element *T_x_*_1_ determines whether it is *OL1* or *OL2*. The *N* state occurs when switching elements *T_x_*_3_, *T_x_*_4_, and *T_x_*_5_ are conducting and switches *T_x_*_1_, *T_x_*_2_, and *T_x_*_6_ are off. In this case, the output voltage is obtained at −0.5 V_dc_ level [25,26].

### 2.2. Field-Oriented Control (FOC)

Field-oriented control is a classic approach preferred for controlling AC machines. This control method is based on the principle of independently controlling torque and flux, similarly to DC motors. The separation of flux and current components is achieved using a rotary coordinate system synchronized with the rotor flux vector. The goal is to achieve high dynamic performance by separating the torque and flux components [27]. To obtain the stator current components required for controlling the PMSM using the FOC method, the abc-αβ transformation is first performed using Equations (1) and (2). The *i_a_* and *i_b_* represent the phase currents *a* and *b* of the stator, while *i_α_* and *i_β_* represent the αβ components of the stator current [2].

(1) $$i_{\alpha }=i_{a}$$

(2) $$i_{\beta }=\frac{1}{\sqrt{3}}i_{a}+\frac{2}{\sqrt{3}}i_{b}$$

After obtaining the αβ components of the current, which are the flux and torque components *i_d_* and *i_q_*, the dq transformation is performed using Equations (3) and (4) [2].

(3) $$i_{d}=i_{\alpha }\cos\theta +i_{\beta }\sin\theta$$

(4) $$i_{q}=-i_{\alpha }\sin\theta +i_{\beta }\cos\theta$$

**Figure 2:**
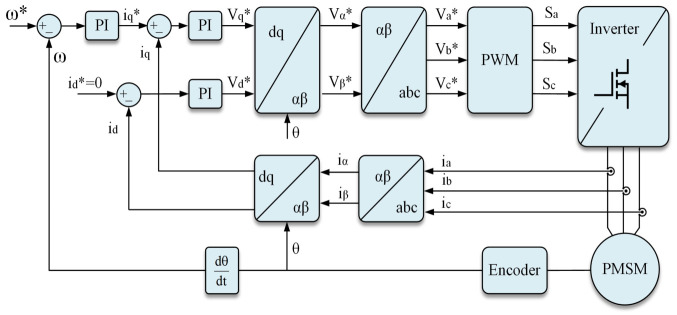
PMSM drive block diagram based on FOC.

The general structure of FOC-controlled PMSM drive with an ANPC inverter is shown in Figure 2 where V_α_* and V_β_* denote the reference voltages in the stationary αβ reference frame while V_a_*, V_b_* and V_c_* represent the corresponding three-phase reference voltages In this control structure, the current and speed information of the PMSM is required to convert the stator currents from the abc plane to the dq axis. The difference between the reference speed value (ω*) and the actual speed value of PMSM is obtained and applied to the PI control input. The reference value of the q component of the stator current is obtained from the PI control output. Then, the difference between the reference d and q current components (i_d_*, i_q_*) and their instantaneous values is applied to the PI controllers to obtain the d and q components of the stator voltage (V_d_*, V_q_*), which are necessary to determine the switching state of the ANPC inverter. The switching signals of the ANPC are obtained using PWM technique by comparing the voltage components obtained from the output of the PI controllers with the carrier signals [28].

### 2.3. Finite Control Set Model Predictive Current Control (FCS-MPCC)

Interest in model-predictive control methods has been steadily increasing over the past thirty years, becoming the subject of research and development. Although it first emerged in the process industry, its use in power electronics began in the 1990s. Its ease of application in multivariate systems requiring rapid dynamic response has led to widespread preference in many fields. Nested control loops can be combined into a single loop using the MPC method. In power electronics applications, control methods are expected to respond in the microsecond range. The disadvantage of the MPC method is its higher computational load compared to other control methods. However, this disadvantage has been overcome with the increased processing power of modern microprocessors, and the applicability of MPC in power converters, inverters, and motor drives in power electronics has increased [2,29]. In EVs, where PMSMs are widely used, the need for advanced control structures to achieve superior dynamic response is increasing every day. Since the current and speed loops in the classical FOC method are PI-based, their limited bandwidth and fixed parameter structure can make them insufficient for high-performance applications. To overcome this, the MPC method, which has a high dynamic response, uses a mathematical model of the PMSM to predict future current behavior in real time, evaluate it in a cost function, and determine the appropriate switching signal [30]. The MPC method, which is the most important predictive control technique for power electronics applications, can be divided into two categories: continuous control set MPC and finite control set MPC. While CCS-MPC requires a modulator to generate switching states, FCS-MPC allows switching states to be generated without a modulator stage. In FCS-MPC, a finite number of switching states of the power converters are used to solve the optimization problem. Therefore, a discrete-time model of the system must be used to predict the future behavior of the system. Then, switching signals that optimize the predefined cost function are determined. Although FCS-MPC can generate switching signals without a modulator, the variable switching frequency is a disadvantage of this method [14,31]. In this study, the FCS-MPCC method, one of the FCS-MPC based control methods, was developed and its performance analysis was carried out in order to provide current control of PMSMs used in electric vehicle propulsion systems.

In the dq-axis coordinate system, the voltages of the three-phase PMSM are expressed by Equations (5) and (6).

(5) $$V_{d}=R_{s}i_{d}+L_{d}\frac{di_{d}}{dt}-\omega_{e}L_{q}i_{q}$$

(6) $$V_{q}=R_{s}i_{q}+L_{q}\frac{di_{q}}{dt}+\omega_{e}L_{d}i_{d}+\psi_{f}\omega_{e}$$

where *L_d_* and *L_q_* are the dq-axis inductances, *R_s_* is the stator resistance, *V_d_* and *V_q_* are the dq-axis components of the PMSM stator voltage, *i_d_* and *i_q_* are the dq-axis components of the stator current, *Ψ_f_* is the permanent magnet flux, and *ω_e_* is the electrical angular velocity of the PMSM [14,32].

The electromagnetic torque (*T_e_*) of the PMSM is calculated using Equation (7), which depends on the dq-axis component of the stator current, the number of pole pairs (*p_p_*), and the permanent magnet flux [32].

(7) $$T_{e}=\frac{3}{2}p_{p}(\psi_{f}i_{q}+(L_{d}-L_{q})i_{d}i_{q})$$

The Euler approximation method for estimating the next values of the stator current with respect to the sampling time *T_s_* is expressed by Equation (8) [32].

(8) $$\frac{dx}{dt}\approx \frac{x(k+1)-x(k)}{T_{s}}$$

The next values of the stator currents of the PMSM are estimated using the Euler method with Equations (9) and (10), where *K*_1_ = *T_s_*/*L_d_* and *K*_2_ = *T_s_*/*L_q_* [33].

(9) $$i_{d}(k+1)=\left(1-K_{1}R_{s}\right)i_{d}(k)+K_{1}L_{q}\omega_{e}(k)i_{q}(k)+K_{1}V_{d}(k)$$

(10) $$i_{q}(k+1)=\left(1-K_{2}R_{s}\right)i_{q}(k)-L_{d}\omega_{e}(k)K_{2}i_{d}(k)+K_{2}V_{q}(k)-\omega_{e}(k)K_{2}\psi_{f}$$

Equation (11) represents the cost function (*g*) generated to control the PMSM’s current using the FCS-MPCC method. The ANPC multilevel inverter has 27 different voltage vectors depending on its switching states, and the dq components of the stator current are estimated for each voltage vector at the next step. These estimated components and reference values are evaluated in the cost function. After evaluating all voltage vectors in the cost function, the switching state that minimizes the cost function is identified. By applying the switching state that minimizes the cost function to the ANPC inverter, the PMSM is made to follow the determined reference current values [2,34].

(11) $$g={\left|i_{d}^{*}-i_{d}(k+1)\right|}^{2}+{\left|i_{q}^{*}-i_{q}(k+1)\right|}^{2}$$

**Figure 3:**
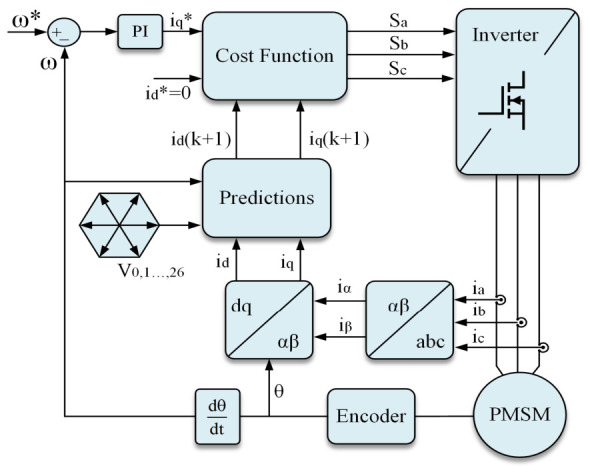
PMSM drive block diagram based on FCS-MPCC control.

In this study, the drive and FCS-MPCC control structure developed to control the PMSM with a three-level ANPC inverter is shown in Figure 3. The difference between the instantaneous speed and the reference speed is applied to the PI controller. The reference value of the q component of the stator current, which is necessary to minimize the speed error, is obtained from the output of the PI controller.

The next values of the dq axis components of the stator currents are predicted using Equations (9) and (10). The differences between the predicted values and the reference stator current values are evaluated in the cost function given in Equation (11). The switching conditions belonging to the voltage vector that minimizes the cost function are determined and applied to the ANPC inverter. Thus, the necessary switching conditions for controlling the PMSM are obtained [2].

## 3. Implementation of FOC and FCS-MPCC-Based PMSM Drives

**Figure 4:**
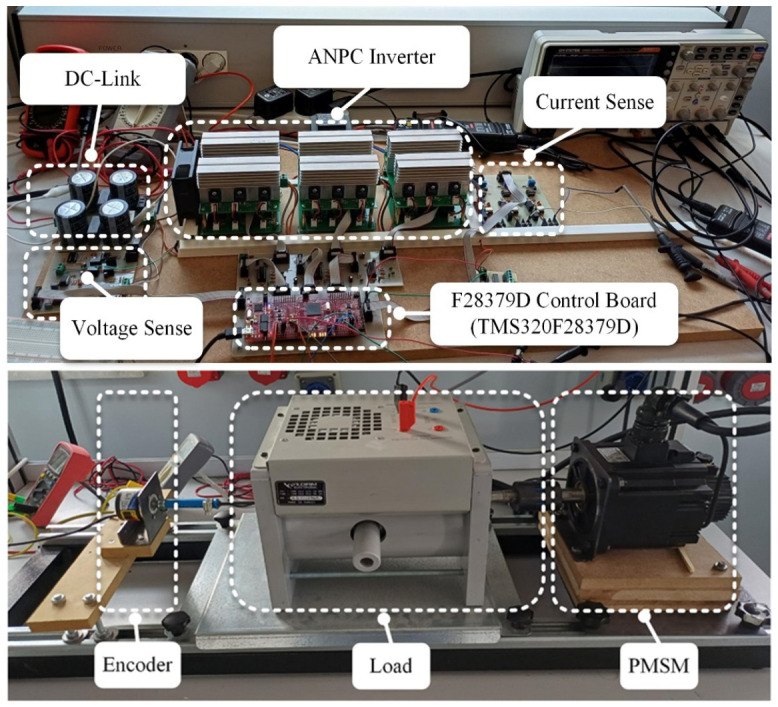
Experimental test bench for PMSM control.

The experimental setup designed in a laboratory environment to realize the real-time performance of the FOC and FCS-MPCC algorithms that are developed to control the PMSM is shown in Figure 4. This experimental setup consists of three main parts: the ANPC inverter, control unit, and auxiliary circuits. The ANPC multilevel inverter topology, which provides higher quality power than classical two-level inverters, was preferred to provide a voltage suitable for the PMSM.

The designed ANPC inverter circuit consists of two layers. The upper layer is comprised by the switching elements, while the lower layer contains an isolated driver layer for each switching element. Isolated sensor circuits were used to obtain the voltage and current data that are necessary for the implementation of the control methods. The Texas Instruments TMS320F28379D DSP development board was used to code the developed FOC and FCS-MPCC algorithms. The FOC and FCS-MPCC algorithms were run separately in real time with this board, and the PMSM was controlled using voltage, current, and speed feedback data. The switching signals required to meet the control conditions were generated by the control board and applied to the driver unit of the ANPC inverter. The dynamic behavior of the system was analyzed for each control algorithm under varying conditions.

Analysis of the FOC and FCS-MPCC methods, whose general structure is given in Figure 2 and Figure 3, was carried out with the designed PMSM drive. The parameters of the PMSM used in the application phase are given in Table 2, and the PMSM drive input voltage was determined as 150 V. The sampling period of the FCS-MPCC algorithm is 200 µs (5 kHz), while the switching frequency in the FOC method is set to 5 kHz.

**Figure 5:**
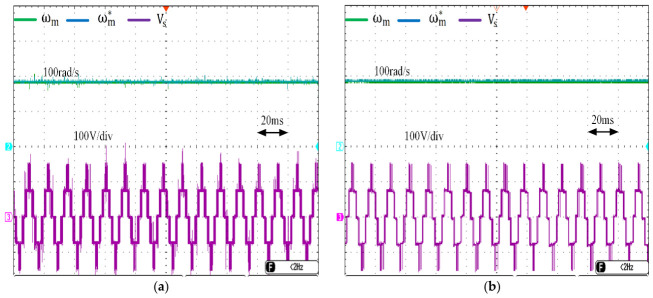
Experimental results of constant reference speed analysis of PMSM: (**a**) FOC; (**b**) FCS-MPCC.

**Figure 6:**
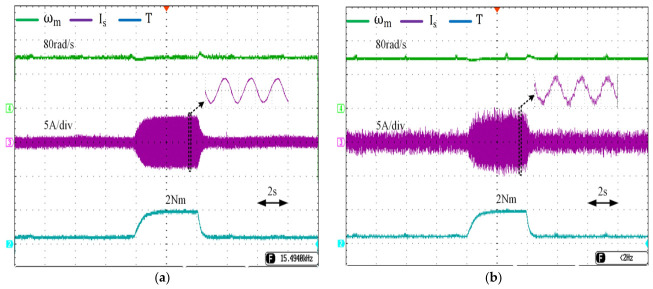
Results of PMSM when the 2 Nm load is applied: (**a**) FOC; (**b**) FCS-MPCC.

Analysis of the FOC and FCS-MPCC methods developed for controlling PMSM was carried out using the developed experimental setup. Initially, the reference mechanical rotor speed (ω_m_*) of the PMSM was set at 100 rad/s for both control methods. Figure 5 shows that the reference speed tracking of the PMSM was successfully achieved with both control methods. The appropriate voltage required for the PMSM was provided by the three-level ANPC inverter, and it was determined that the voltage levels were formed and the peak value was equal to the input voltage of 150 V. Load analyses were performed on the developed FOC and FCS-MPCC-controlled PMSM drives. The reference speed was set at 80 rad/s, and the load of 2 Nm was applied to the PMSM. Figure 6a,b show that the determined reference speed value was followed by both developed control methods. It was also determined that the required torque value was produced by the FOC and FCS-MPCC-controlled PMSM when the load of 2 Nm was applied. In this case, the stator current values of the PMSM are shown, and it was observed that their values were approximately 3.60 A. The FCS-MPC method with variable switching frequency has been observed to have higher current ripple compared to the FOC with fixed switching frequency.

**Figure 7:**
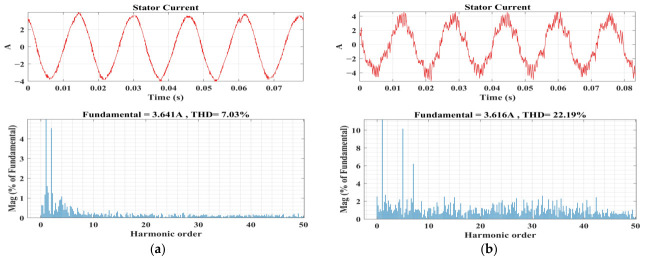
PMSM stator current THD analysis: (**a**) FOC; (**b**) FCS-MPCC.

Figure 7a,b present the harmonic analysis results of the stator currents for both the FOC and FCS-MPCC-controlled PMSM drives. The acquired stator current waveforms were imported into MATLAB/Simulink 2022b for THD analysis. At a reference speed of 80 rad/s under a 2 Nm load, the stator current THD was found to be 7.03% for the FOC-controlled drive and 22.19% for the FCS-MPCC-controlled drive, which indicates the clear advantage of the FOC method in terms of harmonic content. In the FCS-MPCC method, the fifth-order harmonic relative to the fundamental component is determined to be approximately 10 percent. This is one reason high harmonics are observed in the FCS-MPCC method. The switching frequency of the FOC method was fixed at 5 kHz, while the average switching frequency of the FCS-MPCC method was calculated as 1.35 kHz. It appears that THD ratios are significantly affected by the switching methods used in the control structure. This control is attributed to the fixed-frequency PWM modulator embedded within the FOC structure, which inherently produces cleaner current waveforms. In contrast, FCS-MPCC operates with a variable switching frequency, as it selects the voltage vector that minimizes the cost function at each sampling instant, which leads to a broader and less predictable harmonic spectrum. Based on these outcomes, the FOC method demonstrates significantly better harmonic performance than FCS-MPCC. Current harmonics depend not only on the inverter structure but also on the control algorithms. In the FCS-MPCC method, due to variable switching, the ANPC topology cannot completely eliminate harmonic components.

**Figure 8:**
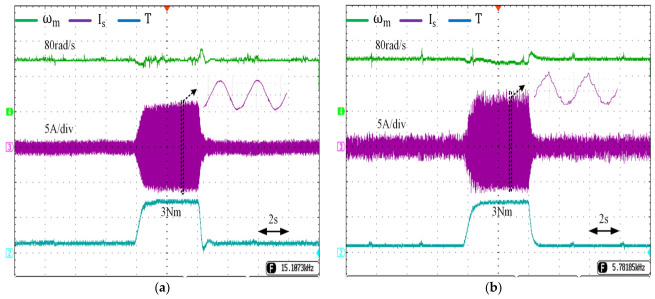
Results of PMSM when the 3 Nm load is applied: (**a**) FOC; (**b**) FCS-MPCC.

Furthermore, the responses of both developed control methods under different loads were analyzed. As shown in Figure 8, the load of 3 Nm was applied to the PMSM instantaneously while the reference speed value was 80 rad/s. With the application of the load to the PMSM, an increase in stator current occurred in both control methods, and it was observed that the necessary torque for the load was produced. It was also determined that both control methods successfully followed the reference speed during load application.

**Figure 9:**
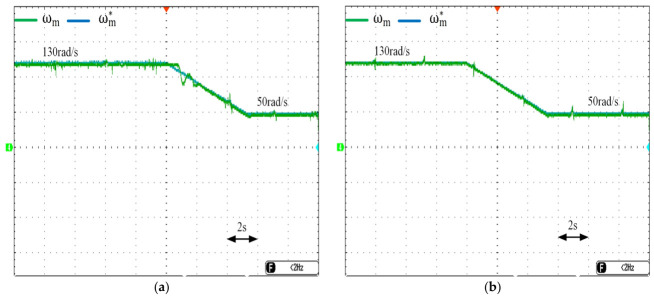
Results across the wide speed range: (**a**) FOC; (**b**) FCS-MPCC.

The performance of both control methods over a wide speed range is shown in Figure 9. The initial reference speed value was set at 130 rad/s and then reduced to 50 rad/s as a ramp. According to the results obtained, it was observed that reference speed tracking was successfully achieved over a wide speed range in both control methods. Although a small transient oscillation occurred during the speed transition in the FOC method, it was determined that the speed tracking was stable. In the FCS-MPCC method, more stable tracking was obtained without this oscillation. According to these results, it is seen that both control methods have the capability of speed tracking over a wide speed range.

**Figure 10:**
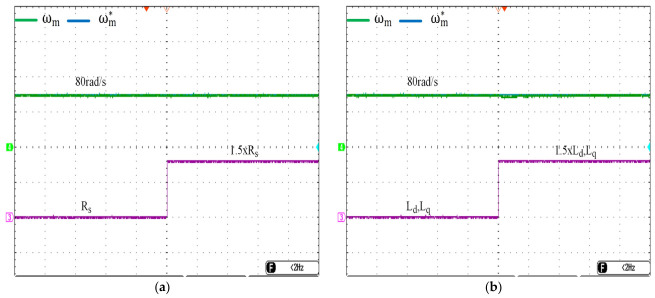
Results of FCS-MPCC parameter variation (**a**) R_s_; (**b**) L_d_, L_q_.

The performance of the FCS-MPCC method was also analyzed when the parameters of the PMSM were varied. Figure 10 shows the success of the FCS-MPCC method when the R_s_ and L_d_, L_q_ parameters were changed individually at a reference velocity of 80 rad/s. It was observed that the FCS-MPCC method, developed to withstand parameter changes, successfully tracked the determined reference velocity. Based on these results, it was determined that the FCS-MPCC method is robust against parameter changes.

Table 3 presents a comparison of the control methods in terms of processing load. The sampling time of the FCS-MPCC algorithm was set to 200 µs (5 kHz), while the switching frequency of the FOC method was set to 5 kHz for a fair comparison of both control methods. The processor loads were calculated using 100 µs, which is the base sample time of the main program files for both control methods. The ANPC-based PMSM drive developed in this study has a total of 27 different voltage vectors. In the FCS-MPCC method, current estimation is performed for these voltage vectors and evaluated in the cost function. The voltage vector that minimizes the cost function is determined and applied to the ANPC inverter. In the FOC method, since it includes the PI controller and PWM modulator, the computational load is lower compared to FCS-MPCC. Therefore, while the processor load is 63.75% in the FCS-MPCC method, it is 17.09% in the FOC method. The fact that the maximum operating values of both control structures are close to the average values indicates that stable performance has been achieved. According to these results, the real-time applicability of the algorithm is demonstrated.

The transient and steady-state performance of the developed FOC and FCS-MPCC controlled PMSM was analyzed in detail under three different reference speed scenarios: step up, step down and triangular.

### 3.1. Scenario 1: The Reference Speed Is Step-Up

**Figure 11:**
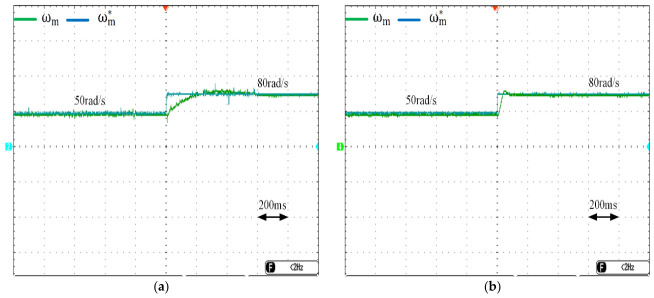
Experimental results of step-up reference speed analysis of PMSM (**a**) FOC; (**b**) FCS-MPCC.

The first scenario involves both control methods by increasing the reference speed value from 50 rad/s to 80 rad/s. This scenario is important for analyzing the dynamic response of the FOC and FCS-MPCC methods developed for PMSM control to this sudden increase in reference speed. The results obtained when the reference speed value increased from 50 rad/s to 80 rad/s are shown in Figure 11. It is observed that the PMSM controlled with FCS-MPCC tracks the specified speed step more precisely compared to the FOC.

**Figure 12:**
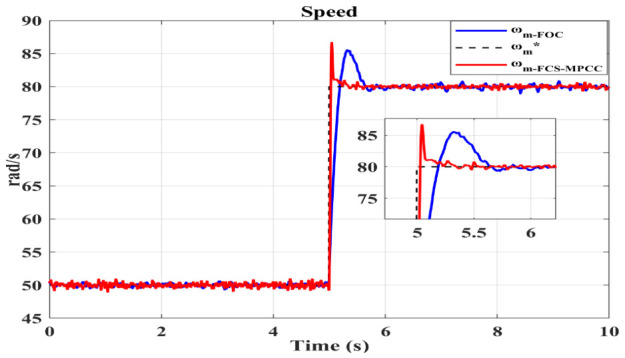
Detailed analysis of step-up reference speed.

Figure 12 shows the detailed MATLAB/Simulink transfer of the speed tracking waveforms obtained with both controllers when the reference speed is increased from 50 rad/s to 80 rad/s. When the reference speed steps from 50 rad/s to 80 rad/s, FCS-MPCC responds faster with a quicker rise time that reaches the new reference sooner. On the other hand, FOC exhibits a larger overshoot that peaks at approximately 85–86 rad/s before settling, while FCS-MPCC also overshoots but settles more rapidly toward the reference value. It is seen that the FCS-MPCC offers faster dynamic response with less overshoot that makes it advantageous during transients. However, FOC delivers better steady-state performance with minimal speed ripple by reflecting the benefit of its fixed-frequency PWM structure.

Table 4 shows that when using the FOC method, the overshoot (*M_p_*) value for the step-up reference speed is 6.90%, while in the FCS-MPCC method, this value is 8.26%.

The rise time (*T_r_*) to the specified reference speed is 0.193 s in the FOC method and 0.039 s in the FCS-MPCC method. Furthermore, the settling time (*T_s_*) to the reference speed is 0.833 s in the FOC method and 0.345 s in the FCS-MPCC method. The higher overshoot exhibited by FCS-MPCC compared to FOC is a side effect of the algorithm’s high bandwidth and dynamic characteristics. The lower overshoot exhibited by FOC relies on the high damping ratio provided by the classical PI control structure, which leads to a longer settling time.

The Mean Absolute Error (MAE), Root Mean Square Error (RMSE), Integral Absolute Error (IAE), Integrated Time Absolute Error (ITAE), and Integral Squared Error (ISE) performance indices given by Equations (12)–(16) are used to determine the dynamic response and stability of control methods [35,36].

(12) $$MAE=\frac{1}{N}\sum_{k=1}^{N}\left|e(k)\right|$$

(13) $$RMSE=\sqrt{\frac{1}{N}\sum_{k=1}^{N}e{\left(k\right)}^{2}}$$

(14) $$IAE=\int \left|e(t)\right|dt$$

(15) $$ITAE=\int t\left|e(t)\right|dt$$

(16) $$ISE=\int e{\left(t\right)}^{2}dt$$

Table 5 presents the analysis results for the MAE, RMSE, IAE, ITAE, and ISE parameters when the reference speed is step-up. In terms of MAE and RMSE performance indicators, the FCS-MPCC method shows approximately 43% improvement compared to FOC. This indicates that the accuracy of the FCS-MPCC method in reference speed tracking is approximately twice as good as that of FOC. Regarding the IAE parameter, it was found that FCS-MPCC has 43.38% lower total error accumulation compared to FOC. This value shows that FCS-MPCC provides more stable reference speed tracking not only in instantaneous errors but throughout the entire operating range. The 48.74% improvement in the ITAE value in the FCS-MPCC method shows that it settles to the reference speed much faster, along with reducing the reference speed tracking error. In terms of ISE, the 67.87% improvement was achieved in the MPCC method.

The lower ISE value obtained with the FCS-MPCC method indicates that it eliminates the reference speed overshoot error much faster than the FOC method and settles back to the reference speed much more quickly. Based on the results, the FCS-MPCC method was found to exhibit better performance in speed tracking compared to the classical FOC method.

### 3.2. Scenario 2: The Reference Speed Is Step-Down

**Figure 13:**
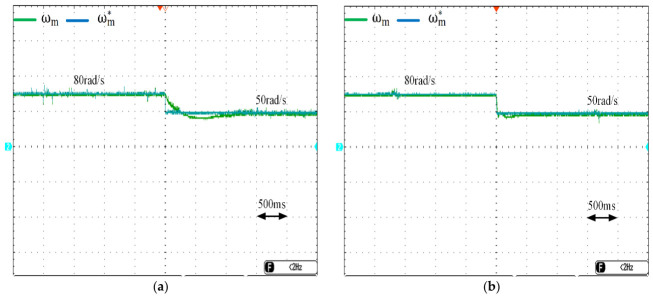
Experimental results of step-down reference speed analysis of PMSM (**a**) FOC; (**b**) FCS-MPCC.

The analyses of the developed FOC and FCS-MPCC methods were performed in the second scenario where the reference speed was step-down. The reference speed value was initially set at 80 rad/s and then reduced to 50 rad/s. In this case, it is shown in Figure 13 that the PMSM performs reference speed tracking when both controllers are used.

**Figure 14:**
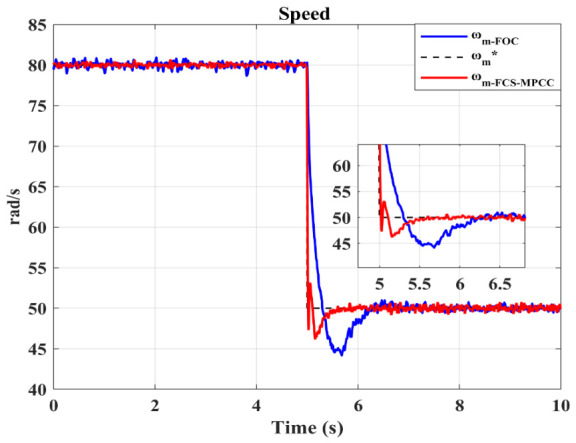
Detailed analysis of step-down reference speed.

In the second scenario, the obtained speed curves were transferred to the MATLAB/Simulink software, and their detailed form is shown in Figure 14. In the case of a step-down reference speed, the undershoot (*M_u_*) value was calculated as 11.65% in the FOC method, while this value was 7.60% in the FCS-MPCC method, as shown in Table 6.

When using the FOC method, the settling time to the determined reference speed (*T_f_*) was 0.3 s, while this value was determined to be 0.019 s in the FCS-MPCC method. When comparing the two control methods in terms of T_s_, it was observed that it was 1.154 s in the FOC method and 0.46 s in the FCS-MPCC method. In the second scenario, the FCS-MPCC method is seen to be more stable than FOC. It is observed that both controllers track the 80 rad/s reference accurately at steady-state operation before the step-down. As observed previously, FOC displays higher speed ripples during steady state, while FCS-MPCC maintains a smoother and more stable speed profile. At the transient response, the FCS-MPCC tracks the declining reference more closely and with a smaller undershoot by dropping to approximately 47–48 rad/s before recovering. On the other hand, the FOC method exhibits a significantly deeper undershoot by falling to nearly 44–45 rad/s that indicates a more aggressive and less damped transient response during deceleration. FOC clearly produces a larger undershoot compared to FCS-MPCC, as is distinctly visible in the zoomed view. This suggests that FCS-MPCC handles the step-down transient more smoothly with tighter control over the speed deviation. FCS-MPCC recovers and stabilizes at the 50 rad/s reference more quickly. FOC takes a longer time to settle, exhibiting oscillatory behavior before converging to the new reference as shown in the inset between approximately 5 s and 6.5 s. Once both methods settle, FCS-MPCC again achieves a smoother steady-state speed with lower ripple, while FOC continues to exhibit comparatively higher ripple around the 50 rad/s reference.

This step-down scenario reinforces the earlier observations. FCS-MPCC demonstrates better transient performance with smaller undershoot and faster settling that makes it more responsive during dynamic speed changes. While producing cleaner steady-state behavior, FOC is more prone to larger deviations and longer settling times during transient events.

When the reference speed curve is step-down, analyses are performed with the speed curves obtained in both control methods in terms of MAE, RMSE, IAE, ITAE, and ISE parameters, and these are presented in Table 7. According to the results obtained, the FCS-MPCC method is seen to perform better than the FOC method in terms of performance indicators. Approximately 61% improvement was obtained with the FCS-MPCC method in terms of MAE, IAE, and ITAE parameters. Accordingly, it shows that the instantaneous sensitivity and time-weighted tracking success of the FCS-MPCC method are superior to the FOC method. The 82.14% improvement obtained according to the ISE criterion shows that the transient damping speed of the FCS-MPCC method is much better than that of FOC. It was determined that the dynamic response of the FCS-MPCC method in minimizing the cost function is high when the reference speed value is step-down.

### 3.3. Scenario 3: The Reference Speed Is Triangular

**Figure 15:**
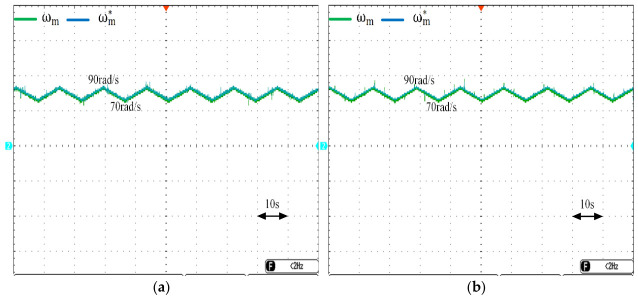
Experimental results of triangular reference speed analysis of PMSM: (**a**) FOC; (**b**) FCS-MPCC.

In the third scenario, the performance of the control methods was evaluated when the reference speed was triangular. Figure 15 shows that speed tracking was possible with both control methods when the reference speed was in a triangular shape varying between 90 rad/s and 70 rad/s.

**Figure 16:**
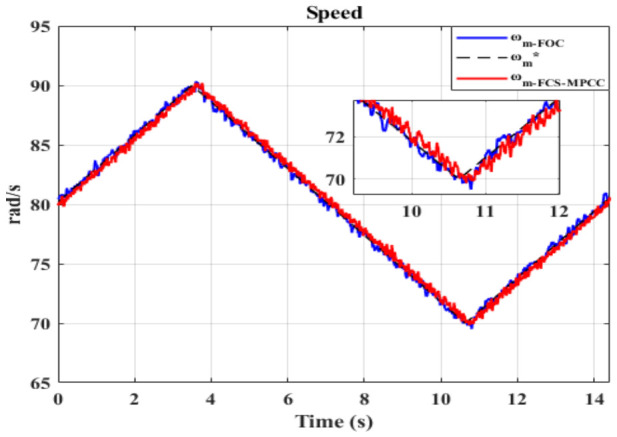
Detailed analysis of triangular reference speed.

The performance analysis of the developed FOC and FCS-MPCC control methods, when the reference speed value is in the triangular form varying between 90 rad/s and 70 rad/s, is detailed in Figure 16. The reference speed follows a triangular profile that varies between approximately 70 rad/s and 90 rad/s over the simulation period. This type of continuously changing reference presents a more demanding tracking challenge compared to step changes, as the controllers must continuously adapt to a varied target. In this scenario, it is observed that both FOC and FCS-MPCC track the triangular reference closely throughout the entire simulation.

Neither method exhibits significant phase lag or amplitude deviation, indicating that both controllers possess adequate bandwidth to follow the slowly varying triangular command. The performance analysis in terms of error criteria, based on the obtained speed curves, is given in Table 8. The FCS-MPCC method shows 39.13% improvement in the ISE value based on the square of large errors compared to FOC that indicates FCS-MPCC is more successful in suppressing overshoots and deviations occurring in transient conditions.

In terms of ITAE values, the 20.11% reduction obtained when using the FCS-MPCC method indicates that the system settles to the reference speed value faster and with greater stability. The approximately 20% improvement in MAE and RMSE parameters indicates that the FCS-MPCC method has better steady-state accuracy.

**Figure 17:**
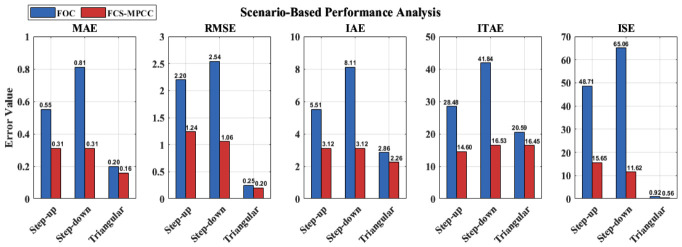
Scenario-based performance analysis.

In this study, speed tracking performance analysis of PMSM control using FOC and FCS-MPCC methods was performed in three different scenarios. According to the data obtained from the experimental results, the FCS-MPCC method exhibited better performance than FOC in terms of MAE, RMSE, IAE, ITAE, and ISE indices, as seen in Figure 17 for all scenarios. The FOC method can cause delays because it includes the cascaded PI controller. When the PMSM is controlled with the FCS-MPCC method, the future state of the system is predicted for each switching period, and the optimum switching state is determined, minimizing errors in the three different analysis scenarios.

According to the results obtained in this study, the dynamic response of the FCS-MPCC method outperforms that of FOC, as determined in Table 9. The FOC method achieved better performance in terms of harmonics due to the constant switching frequency and demonstrated better steady-state performance in terms of lower speed ripple and harmonic content. Furthermore, the FOC method exhibited lower current ripple. In the determined reference speed tracking analyses, it was concluded that the FCS-MPCC method achieved more stable speed tracking according to error criteria.

The dynamic response of FCS-MPCC is better than that of FOC, as confirmed by its smaller overshoot and undershoot during speed step-up and step-down transients, respectively, along with its faster settling to the reference speed. In terms of processing load, the FCS-MPCC method was found to require a higher processing load compared to the FOC method.

## 4. Conclusions

This study presents a performance comparison between FOC and FCS-MPCC control strategies for PMSM drives that are commonly employed in EV applications. An ANPC inverter adopted as a novel multilevel inverter topology was designed to supply the required voltage to the PMSM. Both control methods were implemented and tested in real time under identical conditions, and evaluated across several performance criteria including dynamic response, reference speed tracking, current harmonics, and processor load. Based on the results obtained from speed tracking analysis in three different scenarios in terms of performance criteria MAE, RMSE, IAE, ITAE, and ISE, the FCS-MPCC method was observed to be superior in terms of dynamic performance. The THD ratio of the FOC-based PMSM driver was calculated as 7.03%, while that of the FCS-MPCC-based driver was obtained as 22.19%. The variable switching frequency nature of FCS-MPCC leads to higher current ripple and elevated THD values. In contrast, FOC operates at a fixed switching frequency that results in lower current ripple and a more stable current waveform. When both control methods were analyzed in terms of processor load, the FOC method was found to have a processor load of 17.09%, while the FCS-MPCC method had a load of 63.75%. The FCS-MPCC demands greater computational resources and a longer execution time compared to FOC. In summary, FCS-MPCC proves more advantageous in applications where dynamic response and reference tracking are the primary concerns, whereas FOC demonstrates better performance with respect to processor load and current harmonic content. Future work will focus on investigating and implementing improvements to the FCS-MPCC method, particularly targeting reductions in computational burden and current harmonics.

## Figures and Tables

**Table 1:** Three-level ANPC inverter switching states.

States		Tx1	Tx2	Tx3	Tx4	Tx5	Tx6	Vout
P		1	1	0	0	0	1	0.5 Vdc
O	OU1	0	1	0	1	1	0	0
	OU2	0	1	0	0	1	0	0
	OL1	1	0	1	0	0	1	0
	OL2	0	0	1	0	0	1	0
N		0	0	1	1	1	0	−0.5 Vdc

**Table 2:** PMSM parameters used in the experimental study.

Parameter	Value	Parameter	Value
Power (*P*)	1 kW	Stator resistance (*R_s_*)	0.77 Ω
Number of poles (*p_p_*)	5	d and q-axis inductance (*L_d_*, *L_q_*)	5.09 mH
Nominal speed (*ω_n_*)	209 rad/s	Moment of inertia (*J*)	10.51 × 10^−4^ kgm^2^
Nominal torque (*T_n_*)	4.78 Nm	PM flux (*Ψ_f_*)	0.104 Wb

**Table 3:** Comparison of control methods in terms of processing load.

Control	Avg. Exec. Time	Avg. CPU Load	Max. Exec. Time	Max. CPU Load
FOC	17.09 µs	17.09%	17.14 µs	17.14%
FCS-MPCC	63.75 µs	63.75%	63.76 µs	63.76%

**Table 4:** Dynamic response analysis under step-up reference speed conditions.

Controller	Tr (s)	Ts (s)	Mp (%)
FOC	0.193	0.833	6.90
FCS-MPCC	0.039	0.345	8.26

**Table 5:** Performance indicators when the reference is step-up.

Parameter	FOC	FCS-MPCC	Improvement (%)
MAE	0.55	0.31	43.64
RMSE	2.20	1.24	43.64
IAE	5.51	3.12	43.38
ITAE	28.48	14.60	48.74
ISE	48.71	15.65	67.87

**Table 6:** Dynamic response analysis under step-down reference speed conditions.

Controller	Tf (s)	Ts (s)	Mu (%)
FOC	0.3	1.154	11.65
FCS-MPCC	0.019	0.46	7.60

**Table 7:** Performance indicators when the reference is step-down.

Parameter	FOC	FCS-MPCC	Improvement (%)
MAE	0.81	0.31	61.73
RMSE	2.54	1.06	58.27
IAE	8.11	3.12	61.53
ITAE	41.84	16.53	60.49
ISE	65.06	11.62	82.14

**Table 8:** Performance indicators when the reference is triangular.

Parameter	FOC	FCS-MPCC	Improvement (%)
MAE	0.20	0.16	20
RMSE	0.25	0.20	20
IAE	2.86	2.26	20.98
ITAE	20.59	16.45	20.11
ISE	0.92	0.56	39.13

**Table 9:** Comparison of FOC and FCS-MPCC control methods.

Parameter/Control	FOC	FCS-MPCC
Dynamic response	Medium	Fast
Switching Frequency	Constant	Variable
THD	Low	High
Speed tracking error	Medium	Low
Computational load	Low	High
Current ripple	Low	High

## Data Availability

The raw data supporting the conclusions of this article will be made available by the authors on request.

## Abbreviations

The following abbreviations are used in this manuscript:

CCS-MPC	Continuous Control Set MPC
DTC	Direct Torque Control
EV	Electric Vehicles
FCS-MPCC	Finite Control Set-Based Model Predictive Current Control
FOC	Field-Oriented Control
IAE	Integral Absolute Error
ISE	Integral Squared Error
ITAE	Integrated Time Absolute Error
MAE	Mean Absolute Error
MPC	Model Predictive Control
NPC	Neutral Point Clamped
PMSM	Permanent Magnet Synchronous Machines
PWM	Pulse Width Modulation
RMSE	Root Mean Square Error
THD	Total Harmonic Distortion
VSI	Voltage Source Inverters
